# The Interplay Between Prolactin and Reproductive System: Focus on Uterine Pathophysiology

**DOI:** 10.3389/fendo.2020.594370

**Published:** 2020-10-09

**Authors:** Renata S. Auriemma, Guendalina Del Vecchio, Roberta Scairati, Rosa Pirchio, Alessia Liccardi, Nunzia Verde, Cristina de Angelis, Davide Menafra, Claudia Pivonello, Alessandro Conforti, Carlo Alviggi, Rosario Pivonello, Annamaria Colao

**Affiliations:** ^1^Dipartimento di Medicina Clinica e Chirurgia, Sezione di Endocrinologia, Università Federico II di Napoli, Naples, Italy; ^2^Dipartimento di Medicina Clinica e Chirurgia, Sezione di Endocrinologia, Unità di Andrologia e Medicina della Riproduzione e Sessualità Maschile e Femminile (FERTISEXCARES), Università Federico II di Napoli, Naples, Italy; ^3^Department of Neuroscience, Reproductive Science and Odontostomatology, University of Naples Federico II, Naples, Italy; ^4^Unesco Chair for Health Education and Sustainable Development, “Federico II” University, Naples, Italy

**Keywords:** prolactin, uterus (pathology), uterine cancer, endometriosis, prolactinoma, fertility, dopamine agonist therapy

## Abstract

Over the last years, increasing evidence has focused on crucial pathogenetic role of PRL on malignant, premalignant and benign uterine diseases. Studies in animals and humans have documented that PRL receptors (PRL-Rs) are widely expressed on uterine cells and that PRL is directly synthesized by the endometrium under the stimulatory action of progesterone. Uterine PRL secretion is finely modulated by autocrine/paracrine mechanisms which do not depend on the same control factors implied in the regulation of PRL secretion from pituitary. On the other hand, PRL is synthesized also in the myometrium and directly promotes uterine smooth muscle cell growth and proliferation. Therefore, PRL and PRL-Rs appear to play an important role for the activation of signaling pathways involved in uterine cancers and preneoplastic lesions. Circulating PRL levels are reportedly increased in patients with cervical or endometrial cancers, as well as uterine premalignant lesions, and might be used as discriminative biomarker in patients with uterine cancers. Similarly, increased PRL levels have been implicated in the endometriosis-induced infertility, albeit a clear a causative role for PRL in the pathogenesis of endometriosis is yet to be demonstrated. This evidence has suggested the potential application of dopamine agonists in the therapeutic algorithm of women with malignant, premalignant and benign uterine lesions. This review focuses on the role of PRL as tumorigenic factor for uterus and the outcome of medical treatment with dopamine agonists in patients with malignant and benign uterine disease.

## Introduction

Besides lactotroph cells of anterior pituitary gland (1), prolactin (PRL) is synthesized also in multiple non-pituitary sites including endometrium and myometrium (2). PRL biological actions include beginnings and maintenance of lactation, implantation of pregnancy, proliferation and differentiation of mammary glands cells, immunoregulation and angiogenesis (3). Such biological effects of PRL are mediated by the interaction with prolactin receptors (PRL-Rs). Prolactin receptor (PRL-R) is a transmembrane protein of the cytokine/hemopoietin receptor superfamily, that is encoded by a single gene located on chromosome 5. PRL-Rs are ubiquitous as they are expressed on gonads, uterus, breast, liver, kidney, adrenal gland, brain, heart, pituitary, skin and immune system cells. Binding of PRL to its receptors activates transduction pathways such as Jak-STAT, and proliferative pathways such as mitogen activated protein kinases (MAPK) and phosphoinositide 3 (PI3K). The best-known physiological stimulus for prolactin secretion is breast suckling, that results in a reduction of dopamine release into portal blood (4) reaching the anterior pituitary gland (5), thus essentially relieving the lactotrophs from tonic inhibition.

## Duality of PRL: Pituitary PRL and Peripheral PRL

PRL plays a key role in the reproductive system. In animals and humans, hypersecretion of PRL leads to inhibition of gonadotropin-releasing hormone (GnRH) secretion and to diminished GnRH receptor response to GnRH, together with a decline in luteinizing hormone (LH) pulse frequency and amplitude (6). Particularly, PRL modulates the reproductive axis at central level by acting on a specific population of hypothalamic arcuate nuclei neurons that express the *Kiss1* gene, which encodes neuropeptides, known as kisspeptins, that are critically involved in reproduction (7). Loss-of-function mutations in the genes encoding kisspeptins or the kisspeptin receptor lead to the disruption of puberty and infertility in both human and animal models. Kiss1-expressing neurons are important mediators of PRL effects on reproduction (7). PRL directly acts on Kiss1-expressing neurons and induces suppression of Kiss1 mRNA expression and kisspeptin secretion, leading to a lower activation of GnRH and gonadotropins secretion (7).

PRL modulates the reproductive axis also at peripheral level, as it plays a direct inhibitory effect on the ovaries, leading to decreased estrogen synthesis because of stimulation of 3β-hydroxy-dehydrogenase catalytic activity. Mechanisms of regulation for peripheral PRL secretion are yet to be fully elucidated. In contrast to pituitary PRL, peripheral PRL has been shown not to be regulated by Pit-1 (8) or TRH (9). Previous studies have shown that PRL-Rs are expressed on animal and human ovaries (10, 11), and synthesis of estrogen and progesterone decreases when human ovarian granulosa cells are exposed to supraphysiologic concentrations of PRL (12). The low plasma progesterone concentrations observed in women with hyperprolactinemia have been attributed to a deficient luteal phase (13) with reduced progesterone secretion that leads to poorly developed endometrium and failure of embryo implantation, so contributing to PRL-induced infertility. The impact of PRL on progesterone secretion is dual: PRL potentiates the steroidogenic effects of luteinizing hormone (LH) in granulosa-luteal cells, and inhibits the 20-hydroxysteroid dehydrogenase enzyme, which inactivates progesterone (14). On the other hand, in the last days of a normal menstrual cycle human endometrium is known to produce PRL that, despite its structural and biological similarity with pituitary PRL, does not depend on the same control factors (15). Secretory endometrium synthesizes PRL by direct action of progesterone (15) which induces decidualization of stromal cells, reaching maximum production in the late luteal phase, and by indirect action of estradiol (16) which prepares endometrium for progesterone action. Therefore, synthesis of endometrial PRL is not correlated with serum PRL (17), but rather to differentiation of stromal cells, that is induced by progesterone in a decidualized endometrium (18). Moreover, PRL synthesis in pituitary and endometrium is different, mainly because these two organs display unsimilar stage of differentiation (18): pituitary is a fully differentiated organ at birth, whereas endometrium is undifferentiated until pregnancy (18). This finding is reinforced by the evidence that in the luteal phase the increase in endometrial PRL is not significantly different between normoprolactinemic and hyperprolactinemic women (16). Altogether, these data suggest an autocrine regulation of PRL production at endometrial level, regardless from serum circulating pituitary PRL.

PRL is directly synthesized also in the myometrium (19). In primary cultures of proliferative phase human hysterectomy specimens, PRL production has been demonstrated to progressively and significantly increase after 24, 72, and 96 h even in absence of stimulation by exogenous estrogens and progesterone (19), thus implying the role of other control factors. Interestingly, the addition of estrogen has been found to enhance and that of progesterone to suppress PRL production, respectively, in contrast to decidualized human endometrium where estrogens and progesterone drive opposite effects on PRL production (19).

**Figure 1:**
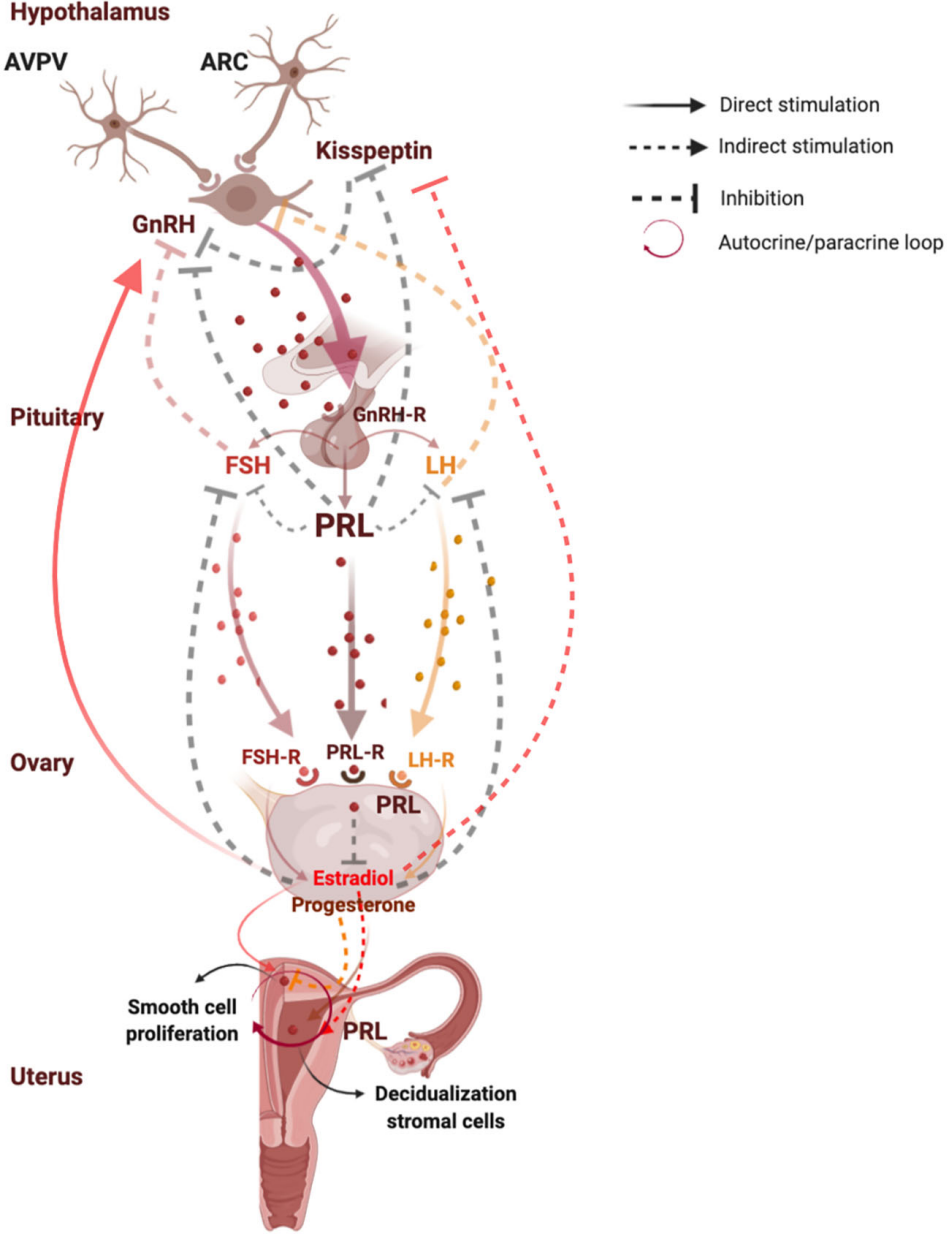
Functions and regulation of pituitary and uterine PRL. PRL plays a key role in the reproductive system, since it modulates the reproductive axis at central level by directly acting on kisspeptins synthesis in the arcuate nuclei neurons where PRL induces suppression of kisspeptin secretion, leading to a lower activation of GnRH and gonadotropins secretion. At peripheral level, PRL plays a direct inhibitory effect on estrogen and progesterone synthesis. In turn, estrogens exert negative feedback on GnRH release on kisspeptin neurons in the ARC, and a positive feedback on GnRH release on kisspeptin neurons in the AVPV. Moreover, in the last days of a normal menstrual cycle human endometrium produces PRL by direct stimulation of progesterone which induces decidualization of stromal cells, and by indirect action of estradiol which prepares endometrium for progesterone action. PRL is directly synthesized also in the myometrium, where it directly stimulates smooth cell proliferation and growth even independently on the stimulation by estrogens and progesterone, suggesting an autocrine/paracrine regulation of uterine PRL synthesis and secretion. AVPV, anteroventral periventricular nucleus; ARC, arcuate nucleus; Kiss 1, kisspeptin 1; GnRH, gonadotropin-releasing hormone; GnRH-R, gonadotropin-releasing hormone receptor; FSH, follicle-stimulating hormone; FSH-R, follicle-stimulating hormone receptor; LH, luteinizing hormone; LH-R, luteinizing hormone receptor; PRL, prolactin; PRL-R, prolactin receptor. Created with BioRender.com.

**Figure 1** shows functions and regulation of pituitary and uterine PRL.

## PRL as a Tumorigenic Promoter: Uterine Malignant and Premalignant Lesions

In several types of cancer, including breast, ovarian, endometrial, and cervical cancers, PRL can have a causal role due to local production or accumulation (20–22). In rodents, hyperprolactinemia has been reported to correlate with increased mammary tumorigenesis (23), and PRL administration has been shown to promote mammary tumorigenesis or tumor growth (24). PRL mRNA was found expressed in human ovarian and endometrial tumor samples (25), and PRL administration was able to stimulate tumor proliferation in five different human endometrial and ovarian carcinoma cell lines (25). PRL-R expression resulted significantly increased in endometrial hyperplasia, as well as in ovarian and endometrial malignant tumors, supporting the presence of an autocrine loop. After chronic exposure to PRL, human immortalized normal ovarian epithelial cells displayed capability to form clones and to grow leading to a malignant transformation (25).

PRL-R has been also found highly expressed either in premalignant lesions or in malignant uterine tumors (26, 27). Studies in cervical cancer cell lines (26) and in paraffin-embedded biopsies from uterine cervical tissue (27), including low grade and high grade squamous intraepithelial lesions and uterine cervical cancers, have reported that PRL-R expression was significantly increased in cervical cancers as compared to preneoplastic lesion or normal tissue samples.

Since the beginning of the 1980s, a relatively high incidence of abnormal serum PRL levels has been reported in women with cervical carcinomas, and the use of dopamine agonists as adjunctive treatment for cervical cancer has been hypothesized. In surgical specimens from cervical cancers and normal cervix, the expression of dopamine receptor type 2 has been shown to gradually increase from normal to cancer tissues, and higher dopamine receptor type 2 expression has been found closely associated with cervical cancer progression (28). Worth to note, the potential direct effects of dopamine agonists on PRL synthesis and release in cervical cancers has not been clearly investigated (28). Conversely, clinical experience with dopamine agonists in patients with cervical carcinomas is encouraging. In a series of 18 patients with cervical carcinoma, treatment with the dopamine agonist bromocriptine has been shown to induce disease remission in 28%, stabilization in 17%, and no response in 55% (29). Median survival passed from 10 weeks in women with no response to 28 weeks in patients with stable disease, to over 6 years in those reaching disease remission (29). Ten years later, the ectopic production of PRL by cervical carcinomas has been demonstrated in cultures of cervical carcinomas (30). Cytoplasmic staining for PRL was positive in 45% of cervical carcinoma tissue sections and in none of the surrounding normal cervix tissue (30). Increased serum PRL levels has been reported in approximately one third of patients with cervical carcinoma, PRL being normalized after surgical removal of the tumor (30).

PRL has been also proposed as biomarker for endometrial cancer, as it resulted to be the stronger discriminative biomarker for endometrial cancer with high diagnostic power even in the early stage in women with endometrial cancer I-III stage (31). Similarly to cervical cancer, dopamine receptor type 2 has found expressed in human uterine tumor tissue sections from patients with endometrial cancers (32), and its overexpression has been shown to correlate with poor overall survival in such patients (32). As for cervical cancers, no direct action of dopamine agonists on PRL synthesis and release from endometrial cancers has been documented (32). Circulating PRL levels have been found significantly higher in women with ovarian and endometrial cancer, as well as in patients with family history of ovarian and endometrial cancer, as compared to those with benign pelvic disease and healthy controls (25).

Altogether, these findings have demonstrated that PRL may play a role as tumorigenic promoter in both malignant and premalignant uterine conditions. Indeed, previous evidence has documented the development of endometrial adenocarcinomas in two women with chronic hyperprolactinemia not responsive to medical treatment with bromocriptine (33), and the use of the PRL receptor antagonist G129R, alone or in combination with paclitaxel, has recently offered promising results, as it has been shown to induce cell death in a mouse model of orthotopic uterine cancer by blocking the short form of PRL-R (34).

## PRL as a Tumorigenic Promoter: Uterine Benign Lesions

The pathogenesis of benign uterine diseases, including adenomyosis, leiomyomas and endometriosis, has been related to direct PRL effects. In mice models adenomyosis has been successfully induced by using intrauterine pituitary isografts (35, 36), and a high incidence of adenomyosis has been found associated with elevated circulating PRL levels, leading to the hypothesis that PRL might be implicated in adenomyosis induction, likely together with the participation of ovarian hormones (36). Administration of bromocriptine to mice bearing pituitary isografts has been shown to completely suppress the induction of uterine adenomyosis (37). Also PRL-R mRNA has been found overexpressed in mice (38) and bovine (39) experimental models of uterine adenomyosis.

Additionally, PRL-R – long-form has been identified in myometrial and leiomyoma smooth muscle cells (40, 41). In cultured uterine smooth muscle cells from myometrium and leiomyomas, treatment with exogenous PRL has been shown to significantly suppress by 25% to 30% leiomyoma cell proliferation after 5 and 8 days, whereas treatment with a PRL-neutralizing antibody blocked the action of endogenous PRL secreted by the cultured cells and inhibited both myometrial and leiomyoma cell proliferation by about 30% (41). These findings led to the conclusion that PRL acts *via* an autocrine or paracrine mechanism to modulate growth of uterine smooth muscle cells in a dose-dependent manner: cell growth and proliferation was promoted at low PRL concentrations and inhibited at high PRL concentrations, suggesting a potential role for PRL in the pathogenesis of leiomyomas (41) and raising the question of whether PRL suppression by medical treatment with dopamine agonists might impact clinical outcome of patients with uterine leiomyomas. In a single blind randomized clinical trial in women with symptomatic leiomyoma (42), treatment with cabergoline 0.5 mg weekly for three months significantly reduced menstrual bleeding and size of both the uterus and the largest myoma as compared to the control group, also relieving symptoms, mainly pain (42). However, few case reports (43–45) have documented persistence of hyperprolactinemia following treatment with the dopamine agonists bromocriptine and cabergoline in three women with uterine myomas. In all these patients, resistance to dopamine agonists was diagnosed on the basis of lack of PRL normalization after medical treatment, and reversal of hyperprolactinemia was induced only by hysterectomy, suggesting that in selected cases extra-pituitary sources of PRL excess should be considered in women with proven resistance to dopamine agonists (43–45).

Altogether, these studies have highlighted that PRL finely regulate the pathogenesis of malignant, premalignant and benign uterine diseases, by directly influencing tumorigenesis in cervical and endometrial cancers, as well as cell growth and proliferation in uterine myomatosis likely with an autocrine or paracrine mechanism. Based on this evidence, PRL might be suggested as potential biomarker for malignant and benign uterine disease, and the use of dopamine agonists bromocriptine and cabergoline might be proposed as adjunctive treatment in such patients to help in restraining tumor growth or to alleviate symptoms in those who can preserve uterus (42).

## PRL Impact in Endometriosis

Aside from neoplastic diseases, PRL is thought to be involved also in the pathogenesis of endometriosis, which reportedly exerts a dramatic negative influence on woman fertility. Increased PRL levels have been implicated in the endometriosis-induced infertility, but studies have failed to prove a clear causal relationship between PRL levels and endometriosis, as discordant results have been reported (46–50). PRL-Rs have been found expressed in normal endometrium but not in endometriotic tissue, at least during the mid-late proliferative phase of the menstrual cycle (51), suggesting a differential regulation of PRL-R expression between normal and endometriotic tissue (51). Given the known capability of endometrium to secrete PRL during the normal luteal phase, a direct PRL secretion from endometriosis implants has been hypothesized (46). However, basal and TRH-stimulated PRL levels have been found similar (47) or increased (48) as compared to controls, and no significant correlation has been reported between PRL levels and luteal phase dysfunction (47) or severity of endometriosis (48). Such a discrepancy has been reported also in studies evaluating PRL levels in the peritoneal fluid of infertile women with endometriosis, where PRL levels have been found similar (49) or increased (50). More recently, serum PRL levels have been reported significantly higher in infertile patients with endometriosis as compared to those without endometriosis, and PRL has been proposed as a biomarker for endometriosis diagnosis and severity (52). A PRL level of greater than 17.5 ng/ml has been found to significantly discriminate patients with and without endometriosis, whereas a PRL level of greater than 20.08 ng/ml has been reported to significantly discriminate between mild (stage I-II) and severe (III-IV) endometriosis (52).

Based on these data, hyperprolactinemia appears to exert a modest and uncertain effect in the pathogenesis of endometriosis, but it is clearly implicated in the endometriosis-induced infertility. These findings have raised the question of whether PRL suppression by dopamine agonists might exert beneficial effects on fertility outcome in patients with endometriosis. This hypothesis is reinforced by the evidence that dopamine receptors type 2 are expressed at gene and protein levels in lesions and surrounding healthy endometrium from women with mild and severe endometriosis, as well as in the endometrium from healthy women (53). Based on this evidence, identification and quantification of dopamine receptor type 2 might represent a novel molecular target for the treatment of endometriosis (53). In rat models, dopamine agonists have shown promising results, since bromocriptine (54), cabergoline (54, 55) and quinagolide (56) have been reported to significantly reduce endometriotic loci size and volume (54–56) following one-month treatment, dopamine agonists being as effective as GnRH agonists in inducing the involution of experimental endometriotic implants in rats (55). In women with endometriosis and serum PRL levels of greater than 30 ng/ml, quinagolide 25 to 75 μg/day has been found to reduce the surface of endometriotic lesions by 69.5% after 4 months of treatment (57). Similarly, in a randomized clinical trial of treatment with cabergoline 1 mg/week for 3 months or LHRH agonist 3.75 mg/month for 3 months, at vaginal ultrasound the reduction of endometrioma size has been demonstrated in 64.7% of patients receiving cabergoline and in 21.7% of those treated with LHRH agonist (58), suggesting the potential application of cabergoline in the clinical setting of patients with endometriosis. Cabergoline has been reported to exert such an effect by promoting vascular endothelial growth factor (VEGF) receptor-2 (VEGF-R2) endocytosis in endothelium, so that to prevent the VEGF-VEGFR-2 binding, thus reducing angiogenesis and inhibiting endometriosis (53, 59). Noteworthy, the impact of dopamine agonists on serum circulating PRL or local PRL secretion by endometriotic lesions has been scantly investigated (54–57), and a prolonged and sustained PRL normalization has been documented only in one study following one-month treatment with quinagolide (57).

In patients with prolactinomas, scant evidence has been collected so far and no clear causative role of PRL excess has been demonstrated in the etiopathology of endometriotic lesions *per se*. Some case reports have documented the diagnosis of endometriosis in patients with mild hyperprolactinemia due to pituitary microadenomas (60, 61). However, even considering that hyperprolactinemia may increase angiogenesis and induce endometriotic lesions (60, 61), in both cases the association of endometriosis and prolactinoma might be casual. In fact, studies investigating prevalence and outcome of endometriosis on large series of women with prolactinomas are still lacking. Conversely, as PRL levels reportedly increase in patients with autoimmune diseases (62), and considering the known association between endometriosis and autoimmune diseases (63), a potential impact of endometriosis on serum circulating PRL, rather than of PRL on endometriosis, cannot be excluded.

Altogether, these findings lead to the conclusion that PRL appear to be a useful biomarker of endometriosis-related infertility and that dopamine agonists might be successfully administered to target endometriotic lesions. This evidence provides the rationale for further investigation on the role of PRL in the pathogenesis of endometriosis, as well as on the potential beneficial effects of dopamine agonists for the treatment of endometriosis-related infertility.

## Uterine Disease in Hyperprolactinemic States

Patients with PRL excess, either tumoral and non-tumoral, experience the disruption of eugonadic state leading to hypogonadism and infertility (64). Particularly, hyperprolactinemic women in fertile age display abnormalities in menstrual cycle ranging from oligomenorrhea to amenorrhea (64), often leading to fertility troubles and overt sterility. Treatment goals for patients with hyperprolactinemia include: 1. control of PRL hypersecretion and its clinical consequences, particularly infertility, sexual dysfunction, and osteoporosis; 2. tumor removal and relief of disturbances in vision and cranial nerve function; preservation of the residual pituitary function; and 3. if possible, prevention of disease recurrence or progression (65–67). However, in asymptomatic patients with hyperprolactinemia, there is no absolute requirement to treat (65). Therapy is usually advisable for macroprolactinomas, and in general for the clinical management of signs and symptoms of hyperprolactinemia *per se*, such as decreased libido, menstrual dysfunction, galactorrhea, infertility, hirsutism, and premature osteoporosis (65, 66). Current available treatment options for prolactinomas include surgery, radiation therapy and pharmacotherapy. Particularly, medical therapy with dopamine agonists is indicated as first-line treatment of patients with both microprolactinomas and macroprolactinomas (67), and nowadays cabergoline represents the treatment of choice for hyperprolactinemia given its higher efficacy both in control of PRL excess and tumor shrinkage over bromocriptine (65–68).

Control of hyperprolactinemia by dopamine agonists results in the restoration of ovulatory menstrual cycles and fertility in up to 90% of women, so leading to the occurrence of spontaneous pregnancies (20, 69). Due to the potential teratogenic effects of dopamine agonists, treatment discontinuation is nowadays required as soon as pregnancy is confirmed, although the safety of both bromocriptine and cabergoline in terms of maternal and fetal outcomes has been extensively documented (70, 71).

Besides the beneficial effects on gonadal function and fertility rate, also uterine perfusion and function parallel the decrease in PRL excess following treatment with dopamine agonists. Particularly, in women with non-tumoral hyperprolactinemia treatment with cabergoline 0.5 mg weekly for 3 months has been shown to significantly reduce the resistance of uterine arteries, evaluated as uterine artery pulsatility index, so decreasing vascular resistance and improving uterine perfusion (72). Restoration of physiologic ovarian cycles and normal uterine perfusion might explain the reason why cabergoline could facilitate pregnancy in women with non-tumoral hyperprolactinemia (72).

These findings lead to the conclusion that hyperprolactinemic states negatively impact women fertility due to the unfavorable effects on both ovarian function and uterine perfusion, and limit spontaneous conception and pregnancy rate. Control of PRL excess by medical therapy with dopamine agonists generally results in restoration of eugonadism and conception rate, allowing the occurrence of spontaneous pregnancies in the vast majority of patients.

## Conclusions

Aside from reproduction, PRL exerts direct biological effects on uterine pathophysiology. PRL is implicated in the pathogenesis of malignant, premalignant and benign uterine diseases: it directly impacts cervical and endometrial tumorigenesis and promotes cell proliferation in uterine myomatosis. These actions of PRL are regulated by autocrine or paracrine mechanisms and appear to be dose-dependent, as PRL promotes or suppress growth and proliferation of uterine smooth muscle cells at low or high concentrations, respectively. Serum PRL levels are reportedly increased in patients with cervical and endometrial cancers, so that to be proposed as biomarker for malignant uterine diseases. Similarly, hyperprolactinemia is commonly found in endometrosis and is known to negatively influence fertility and pregnancy rate in women with this disease. Increased PRL levels also alter uterine vascular resistance and perfusion, thus further impairing woman fertility. Altogether, this evidence provides the basis to focus future investigations on the potential beneficial effects of PRL suppression with dopamine agonists, since promising results have been provided in animal and human models. Bromocriptine and cabergoline might be proposed as adjunctive treatment in patients with uterine malignant and premalignant tumors in order to promote tumor shrinkage and relief from symptoms. Likewise, endometriotic lesions might be effectively targeted by treatment with dopamine agonists even with greater effectiveness as compared to LHRH agonists, and in turn dopamine agonists might act as valid therapeutic strategy to improve fertility in women with endometriosis. However, neoplastic changes in the endometrium have been described in some patients with chronic hyperprolactinemia receiving treatment with dopamine agonists, thus challenging the efficacy of such drugs in uterine malignant diseases, at least in some cases. More recently, treatment with the PRL-R antagonist has shown promising results, and future research will clarify the potential application of this therapy in the clinical setting. On the other hand, evidence collected so far in women with chronic hyperprolactinemia did not clearly document an increased prevalence of uterine neoplastic diseases, and studies specifically focusing on uterine PRL synthesis and release following treatment with dopamine agonists are required to better elucidate the burden and the role of peripheral PRL and dopamine agonists therapy on uterine malignancies.

## Author Contributions

RA made substantial contributions to review of literature, acquisition of data, and interpretation of results and wrote the manuscript. GDV, RS, RPir, AL and NV participated to the review of literature, acquisition of data, and interpretation of results. CDA, DM, CP, AC, CA, and RPiv participated in revising critically the manuscript for important intellectual content. All authors provided critical feedback and helped shape the manuscript. ACol gave final approval of the version to be submitted and any revised version. All authors contributed to the article and approved the submitted version.

## Conflict of Interest

The authors declare that the research was conducted in the absence of any commercial or financial relationships that could be construed as a potential conflict of interest.
